# A Sampling Method Considering Body Size for Detecting the Associated Microbes in Plankton Populations: A Case Study, Using the Bloom-Forming Cyanobacteria, *Microcystis*

**DOI:** 10.3390/biology14111493

**Published:** 2025-10-25

**Authors:** Lizhou Lin, Nanqin Gan, Licheng Huang, Lirong Song, Liang Zhao

**Affiliations:** 1 Guangdong Provincial Key Laboratory of Microbial Culture Collection and Application, State Key Laboratory of Applied Microbiology Southern China, Institute of Microbiology, Guangdong Academy of Sciences, Guangzhou 510070, China; 2 Key Laboratory of Algal Biology, Institute of Hydrobiology, Chinese Academy of Sciences, Wuhan 430072, China; 3 Guangdong Environmental Protection Key Laboratory of Microbiology and Regional Eco-Safety, Guangzhou 510070, China; 4 Kunming Dianchi and Plateau Lakes Institute, Kunming 650228, China; 5 Guangzhou Key Laboratory of Subtropical Biodiversity and Biomonitoring, Guangdong Provincial Key Laboratory of Biotechnology for Plant Development, School of Life Sciences, South China Normal University, Guangzhou 510631, China

**Keywords:** group analysis, *Microcystis* associated microbes, high-throughput sequencing, sampling

## Abstract

**Simple Summary:**

It is challenging to accurately measure the microbes living in close association with tiny aquatic organisms. This is because many laboratory methods can only detect the relative amounts of microbes compared to their host, not the absolute number. If the host’s size affects this relative amount, simply testing random individuals may yield misleading results about the whole population. In this study, we simulated a dataset based on a globally widespread phytoplankton to test a different approach called group analysis. This method tests individuals in groups rather than individually. We found that this approach greatly improves accuracy and reduces errors, especially when analyzing groups with many individuals per group. This method also facilitates comparison of different populations and makes it easier to detect microbes present in very small numbers. Our findings offer a practical way to better understand the relationships between small aquatic organisms and their associated microbes, which is crucial for protecting water quality and ecosystem health.

**Abstract:**

Accurately quantifying associated microbes is essential to understand the interactions between microplankton and their associated microbes. Most DNA-based methods, such as high-throughput sequencing, primarily assess the ratio of target objects to references in microplankton samples. However, simple random sampling (SRS) of individuals may lead to deviations in quantifying these ratios at the population level if these characteristics are associated with the reference content of individuals. This study considered group analysis, which involves detecting *k* groups with *n* individuals in each group, as an alternative approach and used simulated data based on the detection of *Microcystis* populations to evaluate the accuracy of different sampling plans. Our results indicate that increasing the number of individuals in each group could reduce sampling bias and improve the accuracy of comparisons between populations. Group analysis could also minimize the impact of the detection limit. This study demonstrated that, when detection methods only provide the ratio of target objects to references, group analysis is more appropriate than SRS for characterizing microplankton populations. Group analysis can be used not only for detecting associated microbes but also for identifying ingested organisms or the biochemical composition of microplankton. Our results also demonstrate how in situ individual-level studies support ecological investigations.

## 1. Introduction

The interactions among planktonic organisms are complex and play an important role in aquatic ecosystems [1,2]. These interactions can be species-specific. For instance, parasitic dinoflagellates could affect the dynamics of harmful algae, copepods, and fish by infecting their hosts in a species-specific mammer [3]. The phycosphere of *Phaeodactylum tricornutum* and *Microchloropsis salina* was found to be associated with distinct bacterial communities [4]. The microbial impact on their hosts also differed: while *Algoriphagus* and *Muricauda* suppressed the biomass of *Phaeodactylum tricornutum*, *Marinobacter* enhanced it [5]. Additionally, some members of certain plankton, such as *Aphanizomenon*, *Microcystis*, *Dolicospermum*, and *Gymnodinium*, have the capability to produce toxins like microcystins or saxitoxin, which are harmful to protozoa, shellfish, and fish, thereby threatening aquatic ecosystems and human health [6,7]. Therefore, to better estimate the ecological impacts of planktonic organisms, it is essential to understand the biochemical composition and associated microbes within certain plankton populations.

Different populations, such as phytoplankton, bacterioplankton, and zooplankton, can coexist in the same habitat. However, it is often challenging to completely separate these populations and assess their characteristics. Therefore, biochemical contents and associated microbes are often detected at the community level [8,9]. The correlations between species revealed by community-level studies may reflect the characteristics of different species but can result from similar niches rather than direct interactions between organisms [10]. Therefore, population-level detection is necessary to verify the properties of a specific population in situ.

Surveys of randomly selected individuals (simple random sampling, SRS) have been used to assess the characteristics of populations. For example, in 1986, Rivkin et al. [11] used Flow Cytometric Analysis to measure the carbon uptake by natural phytoplankton populations. The detection methods for individuals include both qualitative and quantitative approaches. Microscopic examination can directly verify the relationships among microbes, such as the parasitism of *Amoebophrya* on host dinoflagellates and the grazing of *Noctiluca* on algae [12,13,14]. However, microscope-based methods often struggle to quantify associated microbes effectively. Other methods, including MALDI-TOF-MS (matrix-assisted laser desorption/ionization time-of-flight mass spectrometry), single-cell Raman microspectroscopy and DNA-based methods, have been employed for the quantitative or semi-quantitative detection of biochemical content or associated microbes of individuals. For example, PCR has been used to detect microcystin synthesis genes of *Microcystis* colonies [15]. PCR-DGGE of single colonies has been used to reflect the associated bacteria of *Microcystis* colonies in situ [16,17]. MALDI-TOF-MS has been utilized to reflect microcystin content in individual *Microcystis* colonies [18,19].

It is often challenging to figure out the absolute content of biochemicals or microbes in semi-micro or micro samples using these quantitative or semi-quantitative methods (such as PCR-DGGE, MALDI-TOF-MS, or high-throughput sequencing); instead, they typically provide the ratio of specific biochemicals or microbes to reference standards that reflect the biomass of the hosts. References for DNA-based methods could be 16S, 18S rDNA, or PC-IGS gene amplicons [15,18], while chlorophyll derivatives serve as references for MALDI-TOF-MS [18,19]. Single-cell Raman microspectroscopy utilizes ratios, such as C^13^:C^12^, to reflect the activities of cyanobacteria cells [20].

In aquatic ecosystems, the size of aggregates or colonies can significantly influence biological functions. For example, the concentrations of microcystins and extracellular polysaccharides in *Microcystis* colonies depend on colony sizes [21,22]. Additionally, the associated bacterial communities vary among *Microcystis* colonies of different sizes [23]. The anaerobic zones within cyanobacteria aggregates, which are crucial for anaerobic microbes, require a certain aggregate size [24]. Furthermore, the grazing preferences of zooplankton are related to their body size [25]. However, it remains unclear whether the correlation between body size and biological function affects the accuracy of detecting population characteristics.

To determine whether the detected results from sampled individuals accurately reflect the true values in populations, the ratio of bacteria to *Microcystis* within colonies (individuals) has been used. *Microcystis* typically existed as colonies [26], which are commonly associated with microbes in eutrophic freshwater lakes [17]. These associated microbes are believed to play an important role in *Microcystis* colony formation and nitrogen/phosphorus utilization [27,28,29]. The ratios of associated microbes to *Microcystis* at the population level are as follows:

(1) $$R_{T}=\frac{Bac}{M}=\frac{\sum_{i=1}^{t}{Bac}_{i}}{\sum_{i=1}^{t}m_{i}}=\frac{\sum_{i=1}^{t}m_{i}r_{i}}{\sum_{i=1}^{t}m_{i}}$$

*Bac* represents the total biomass of certain bacteria associated with *Microcystis* at the *Microcystis* population level; *M* is the total biomass of the *Microcystis* population; *Bac_i_* is the biomass of certain bacteria associated with the *i*th *Microcystis* colony; *m_i_* indicates the biomass (represented by body size) of the *i*th *Microcystis* colony; *r*_i_ is the ratio of *Bac_i_* to *m_i_*; *t* is the number of colonies within the *Microcystis* population.

The Pearson correlation coefficient (*ρ*) between *Microcystis* and a certain microbe is as follows:

(2) $$\rho =\frac{Cov\left(M,R\right)}{\sqrt{Var\left(M\right)Var\left(R\right)}}=\frac{E\left[MR\right]-E\left[M\right]E\left[R\right]}{\sqrt{Var\left(M\right)Var\left(R\right)}}$$

Then, the ratios of associated microbes to *Microcystis* at the population level can be represented as follows:

(3) $$R_{T}=\frac{\sum_{i=1}^{t}m_{i}r_{i}}{\sum_{i=1}^{t}m_{i}}=\frac{\frac{1}{t}\sum_{i=1}^{t}m_{i}r_{i}}{\frac{1}{t}\sum_{i=1}^{t}m_{i}}=\frac{E[MR]}{E[M]}=\frac{\rho \sqrt{Var\left(M\right)Var(R)}+E\left[M\right]E[R]}{E[M]}=E\left[R\right]+\rho \frac{\sqrt{Var\left(M\right)Var\left(R\right)}}{E\left[M\right]}$$

*E*[*M*], *E*[*R*], and *E*[*MR*] represent the average (expectation) *Microcystis* biomass, ratio of a certain microbe to *Microcystis*, and the arithmetic product of *Microcystis* biomass and the microbe-to-*Microcystis* ratio in individuals, respectively. *Var*(*R*) and *Var*(*M*) are the variances of the ratios and *Microcystis* biomass. *Cov*(*M*,*R*) is the covariance between *Microcystis* biomass and the ratio of a certain microbe to *Microcystis*. If *M* and *R* are independent, then *ρ* = 0, *R_T_* = *E*[*R*]; conversely, if *M* and *R* were correlated, then ρ≠0, $R_{T}\neq E[R]$. This suggests that simple random sampling may yield incorrect estimates of the associated microbe ratio in a *Microcystis* population. Particularly, when ρ>0, *E*[R] < *R_T_*, estimation via simple random sampling is less than the true value. When ρ<0, *E*[R] > *R_T_*, estimation via simple random sampling is larger than the true value.

Additionally, some investigations may focus on the proportions of associated communities [30,31]. The calculation of microbial community composition is as follows:

(4) $$P_{i}=\frac{{Bac}_{i}}{\sum_{i=1}^{N}{Bac}_{i}}=\frac{{Bac}_{i}/M}{\sum_{i=1}^{N}\left({Bac}_{i}/M\right)}=\frac{R_{i}}{\sum_{i=1}^{N}R_{i}}$$

*P_i_* represents the proportion of the *i*th microbe in the community; *R_i_* is the ratio of the *i*th microbe to *Microcystis. Bac_i_* refers to the *i*th microbe, and *M* is *Microcystis*. Therefore, the estimation of community structure is calculated via the ratio of microbes to *Microcystis*, and is influenced by the correlation between *Microcystis* and the ratio of microbes to *Microcystis.*

**Figure 1:**
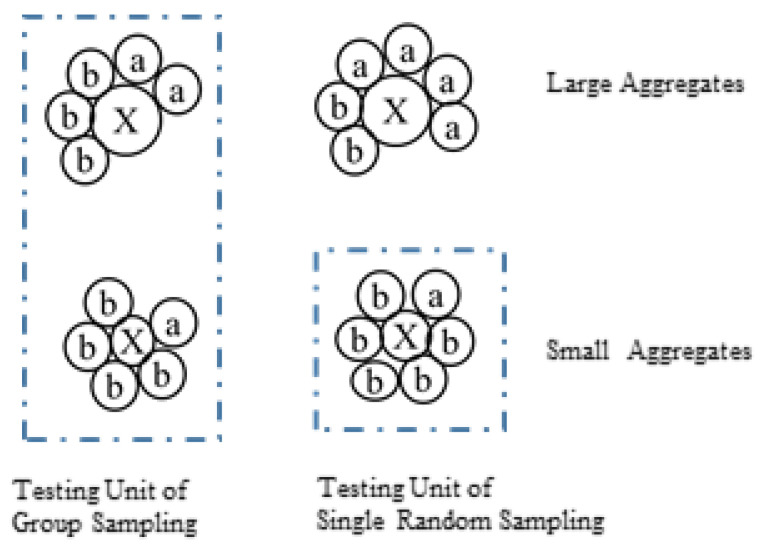
The sampling plans of simple random sampling and group analysis.

In addition to simple random sampling, group analysis provides another option. The group analysis process is conducted as follows: (1) randomly sample *n* individuals to form one group; (2) repeat step (1) to obtain *k* groups; (3) mix the individuals in the same group and test as a whole (Figure 1). The ratio received via group analysis was calculated as follows:

(5) $$R_{C}=\frac{1}{k}\sum_{i=1}^{k}\frac{\sum_{j=1}^{n}m_{ij}r_{ij}}{\sum_{j=1}^{n}m_{ij}}$$

And the ratio received via simple random sampling was calculated as:

(6) $$R_{C}=\frac{1}{kn}\sum_{i=1}^{kn}r_{i}$$

Group analysis is widely employed in the quality control of grain and silkworm eggs, achieving acceptable results [32,33]. The efficiency of group analysis in seed purity testing has been estimated based on the binomial distribution [34,35]. However, the efficiency of group analysis in ecological sampling remains limited. Venrick [36] investigated the use of parallel samples from multiple Sedgwick-Rafter chambers, which shares conceptual similarities with group sampling. However, their focus was on the precision of phytoplankton biomass estimates and the variability among chambers within a single sample, rather than on the relative abundance of microbes to their hosts. In 2016, Ray et al. [25] employed amplicon sequencing to investigate copepod grazing with three replicates, each having five copepod individuals. Nevertheless, the effect of this sampling strategy was not discussed. Therefore, the effectiveness of group analysis requires further assessment, particularly for the quantitative detection of biochemicals and associated microbes within planktonic populations.

The aim of this study was to assess the efficiency of sampling methods, focusing on the accuracy of associated microbe detection at the population level, as well as the efficiency and accuracy of group analysis in comparing two populations. Our experiment involved the following components: (1) constructing the simulated dataset containing a large number of individuals, based on the detected *Microcystis* colony size and associated microbes; (2) evaluating the accuracy of the results achieved via SRS and group analysis; (3) examining the efficiency and accuracy of different sampling plans in comparing the abundance of microbes in two *Microcystis* populations.

## 2. Materials and Methods

### 2.1. Microcystis Colony Size Measurements

*Microcystis* colonies were collected from Dianchi Lake on 22 June 2015. *Microcystis* morphospecies were identified as described by Komárek and Komárková [26]. The colony sizes of *Microcystis wesenbergii* (*M.w.*) and *Microcystis aeruginosa* (*M.a.*) were measured using the image analysis software NIS Elements 4.10, with a Nikon H550L digital camera (Japan), as described by Wilson et al. [37].

### 2.2. Detection of Associated Microbes in Microcystis Colonies

Single colonies of *M.a.* and *M.w.* were selected under the microscope via Pasteur pipettes, then placed into axenic 0.2 mL tubes. Each morphospecies of *Microcystis* colony was placed into at least five tubes, with each tube containing at least 10 randomly selected colonies. The colonies were washed with axenic BG11 cultures at least three times, then stored at −80 °C until DNA extraction. Genomic DNA extraction was performed using a DNA kit (MP Fast DNA SPIN Kit for soil, 116560-200, Irvine, CA, USA) according to the manufacturer’s protocol. Illumina Miseq Sequencing of the 16S rRNA gene was performed using primers (341F: 5′-CCTACGGGNGGCWGCAG-3′, 785R: 5′-GACTACHVGGGTATCTAATCC-3′) targeting the V3 and V4 regions [38]. After removing the low-quality reads via Fastp (V0.21.0), paired-end reads were merged into Clean Tags using vsearch –fastq_mergepairs (V2.19.0) [39]. Then the primers in Clean Tags were removed based on the primer sequences via Cutadapt (V3.4) [40]. Clean Tags were de-replicated into amplicons sequence variants (ASVs) with 100% similarity via dada2 [41] and we obtained the ASV abundance table. The AssignTaxonomy function of dada2 in R 3.6.0 was used for the alignment of Representative sequences of ASVs based on the Silva 16S rRNA gene data base (v138, https://www.arb-silva.de/) (accessed on 16 December 2019). After the taxonomy assignment of ASVs, the read numbers of *Xanthomonadales* and *Microcystis* of each sample were extracted from the ASV abundance table. The ratio of *Xanthomonadales* to *Microcystis* was calculated based on the ASVs, and then used in this study.

### 2.3. Simulated Population Construction

Datasets **X**, **Y**, and **Z** included the colony sizes (**S**^X^, **S**^Y^, **S**^Z^) and the ratios of microbes to *Microcystis*, (**R**^X^, **R**^Y^, **R**^Z^). Each dataset included three sub-datasets, each containing one **S** sequence and one **R** sequence. The correlation between **S** and **R** in the three sub-datasets was, respectively, independent, positively correlated, and negatively correlated. **R** and **S** were lognormally distributed. The characteristics of datasets **X**, **Y** and **Z** were as follows: (1) the sample sizes of **S**^X^, **S**^Y^, **S**^Z^, **R**^X^, **R**^Y^, and **R**^Z^ were 500,000; (2) the average and standard deviation of the logarithm of ***S****^X^* and ***S****^Y^* were equal to those of the logarithm of *M.a.* and *M.w.* colony size; ***S****^Z^* was the same as **S*^X^***; (3) the averages of the logarithm of **R**^X^ and **R**^Y^ were equal to those of the logarithm of *Xanthomonadales*: *Microcystis* in the *M.a.* and *M.w.* colonies, respectively; (4) the correlation coefficient between **R**^Z^ and **S**^Z^ was different from that between **R**^X^ and **S**^X^.

The simulation process for the independent sub-datasets of **X**, **Y**, and **Z** was as follows:

(1) A sequence $N_{S}^{X}$ was created with a length of 500,000, averaging Mean(log(***S****^M.a.^*)), and the standard deviation was SD(log(***S****^M.a.^*)); $Exp(N_{S}^{X})$ was the simulated ***S****^X^*. ***S****^Y^* was calculated in the same step based on Mean(log(***S****^M.w.^*)) and SD(log(***S****^M.w.^*)). **R**^X^ and **R**^Y^ were calculated based on Mean(log(***R****^M.a.^*)) and Mean(log(***R****^M.w.^*)).

The positively or negatively correlated sub-datasets were simulated as follows:

(2) $R_{1}=\left(\sum_{i=1}^{t}S_{i}^{X}R_{i}^{X}\right)/\left(\sum_{i=1}^{t}S_{i}^{X}\right)$, which presents the actual *R_T_* value of the community in this sub-dataset.
(3) $P=BN_{S}^{X}+CN_{R}^{X}$.

$N_{S}^{X}$ and $N_{R}^{X}$ are the normally distributed sequences obtained via step (1). Constants B and C were applied, with C > 0 and B > 0 for the positively correlated ***R***; B < 0 when the negatively correlated ***R*** was simulated.

(4) $A=Exp\{\left[P-Mean\left(P\right)\right]/SD\left(P\right)\times SD(N_{R}^{X})+Mean(N_{R}^{X})\}$. ***A*** is a lognormally distributed sequence based on ***P***.
(5) $R_{2}=\left(\sum_{i=1}^{t}S_{i}^{X}A_{i}\right)/\left(\sum_{i=1}^{t}S_{i}^{X}\right)$. The ***R_T_*** of the community with ***A*** obtained from step (4) was obtained as an ***R_positive_*** sequence and ***S^X^*** as an S sequence.
(6) $R_{positive}^{X}=A/R_{2}\times R_{1}$.

To better compare the impact of the different correlations of **R** and **S** on the sampling results, the three sub-datasets were set as $R_{T,positive}^{X}=R_{T,negative}^{X}=\;R_{T,Independent}^{X}$ at the community level. So, all data in ***A*** was then multiplied by the coefficient R_1_/R_2_.

(7) $R_{negative}^{X}$ was simulated according to steps (2) to (6), with the constants *B* in step (2) being negative.

$R_{positive}^{X}$ was lognormally distributed and positively correlated with ***S****^X^*, while $R_{negative}^{X}$ was lognormally distributed and negatively correlated with ***S^X^***. The ratio *R_T_* calculated based on $R_{positive}^{X}$ and, $R_{negative}^{X}$, and ***S****^X^* was equal to that calculated based on $R_{independent}^{X}$ and ***S****^X^*.

(8) Datasets **Y** and **Z** were simulated in a similar manner.

### 2.4. The Calculation of Microbe/Microcystis via Different Sampling Plans

The sampling plan is denoted by Plan(*n*,*k*), where (1) *n* represents the number of individual colonies in each group and (2) *k* represents the number of testing groups. When *n =* 1, Plan(*n*,*k*) was simple random sampling. The true ratio of microbe/*Microcystis* in the *Microcystis* population (*R_T_*) and the ratio received via Plan(*n*,*k*) (*R_C−n,k_*) were calculated as follows:

(7) $$R_{T}=\frac{\sum_{i=1}^{t}s_{i}r_{i}}{\sum_{i=1}^{t}s_{i}}$$

(8) $$R_{C-n,k}=\frac{1}{k}\sum_{i=1}^{k}\frac{\sum_{j=1}^{n}s_{ij}r_{ij}}{\sum_{j=1}^{n}s_{ij}}$$

*s_ij_* is the size of the *i*th *Microcystis* colony in the *j*th group; *r_ij_* is the microbe/*Microcystis* of the *i*th *Microcystis* colony in the *j*th group.

For each Plan(*n*,*k*), sampling was performed 99 times, receiving 99 values for *R_C−n,k_*. The average and standard deviation of these values (Mean(*R_C−n,k_*) and SD(*R_C−n,k_*)) were calculated to represent the expected value and deviation of *R_C−n,k_* (*E*[*R_C−n,k_*])).

### 2.5. Statistical Efficiency of Different Sampling Plans

The test of significance was based on the permutation of the average microbe/*Microcystis* in different populations, using the coin package in R 3.1.2. When comparing two populations **A** and **B**, the null hypothesis for the permutation test was H_0_: $R_{T}^{A}=R_{T}^{B}$, while the alternative hypothesis were H_1_: $R_{T}^{A}<R_{T}^{B}$ and H_2_: $R_{T}^{A}>R_{T}^{B}$. When *p* > 0.05, the null hypothesis was accepted; when *p* < 0.05, the null hypothesis was rejected. For $Mean(R_{C-n,k}^{A})<Mean(R_{C-n,k}^{B})$ or $Mean(R_{C-n,k}^{A})>Mean(R_{C-n,k}^{B})$, hypothesis H_1_ or H_2_: was accepted, respectively. For each Plan(*n*,*k*), the permutation test was performed 999 times. The probability of accepting H_0_, H_1_ and H_2_ (P(H_0_), P(H_1_) and P(H_2_)) was calculated as the proportion of accepting each hypothesis. In datasets **X** and **Z**, $R_{T}^{X}=R_{T}^{Z}$, H_0_ was correct. For datasets **X** and **Y**, $R_{T}^{X}<R_{T}^{Y}$, H_1_ was correct.

## 3. Results

### 3.1. Distribution of Microcystis Colony Size

**Figure 2:**
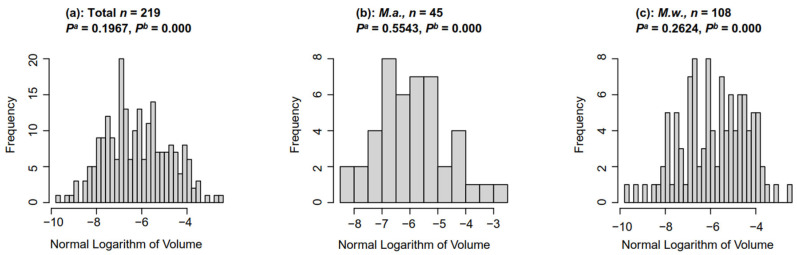
The histogram of the natural logarithm of colony volume (mm^3^). *P^a^*: the significance of the Shapiro–Wilk test based on the normal logarithm of volume; *P*^b^: the significance of the Shapiro–Wilk test based on volume.

The colony sizes of *M.a.* and *M.w*. showed a wide distribution. The colony size of *M.a.* in Dianchi Lake ranged from 0.0003 mm^3^ to 0.07 mm^3^, and that of *M.w.* ranged from 0.0001 mm^3^ to 0.08 mm^3^. The sizes of *Microcystis* colonies collected from Dianchi Lake were tested using the Shapiro–Wilk test. The results showed that the colony sizes of total *Microcystis*, *M.w*., and *M.a.* conformed to lognormal distribution (Figure 2, Shapiro–Wilk, *p* > 0.05) rather than a normal distribution (Figure 2, Shapiro–Wilk, *p* < 0.001).

### 3.2. Characteristics of Simulated Datasets

The datasets **X**, **Y** and **Z** were simulated based on the measurements of *Microcystis* colonies, with the ratios and sizes conforming to lognormal distributions (Table 1, Lilliefors test, *p* > 0.05). The R_T_ values of the sub-datasets within each dataset were equal to one another. The correlation between ***S*** and ***R*** of datasets **X**, **Y**, and **Z** was independent in sub-dataset 1 (Table 1, *p* > 0.05), positive in sub-dataset 2 (Table 1, *R* > 0, *p* < 0.01), and negative in sub-dataset 3 (Table 1, *R* < 0, *p* < 0.01).

### 3.3. Accuracy of Different Sampling Plans

#### 3.3.1. Systematic and Stochastic Errors of Different Sampling Plans

**Figure 3:**
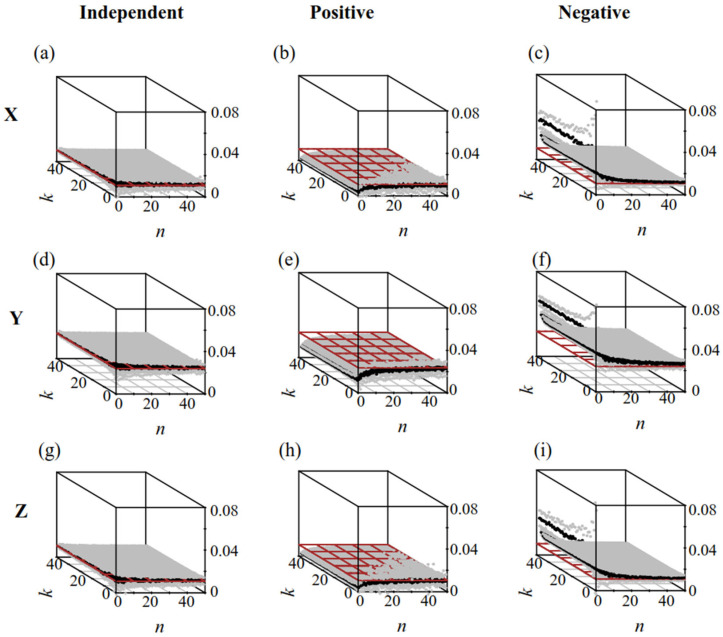
The exacted microbe/*Microcystis* value in populations (*R_T_*) and calculated microbe/*Microcystis* ($R_{C-n,k}$) via Plan(*n*,*k*). Brown plane: *R_T_*; Black nodes: $\mathrm{Mean}(R_{C-n,k})$; Gray nodes: $\mathrm{Mean}(R_{C-n,k})\;\pm \;\mathrm{SD}(R_{C-n,k})$. (**a**–**c**,**d**–**f**,**g**–**i**) were the results of dataset X, Y, and Z, respectively. The *R* and *S* in (**a**,**d**,**g**) were independent. The *R* and *S* in (**b**,**e**,**h**) were positively correlated; The *R* and *S* in (**c**,**f**,**i**) were negatively correlated.

When ***R*** was independent of ***S*** (Figure 3a,d,g), the results of group analysis and simple random sampling were consistent with the actual values: $Mean\left(R_{C-1,k}\right)=Mean\left(R_{C-n,k}\right)=R_{T}$.

**Figure 4:**
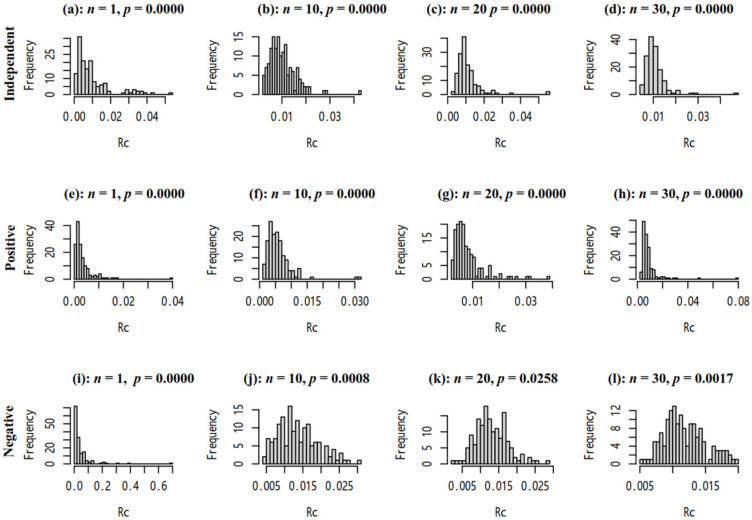
The histogram of *R_C_* via sampling Plan(*n*,150). *P* presents the significance of the Shapiro–Wilk test. (**a**–**d**) Ratio and reference were independent. (**e**–**h**) Ratio and reference were positively correlated. (**i**–**l**) Ratio and reference were negatively correlated.

When ***R*** (microbe/*Microcystis*) was correlated with ***S*** (colony size), simple random sampling affected the systematic errors. Increasing the value of *n* in group analysis reduced the systematic errors caused by sampling (Figure 3b,c,e,f,h,i). When ***R*** was positively correlated with ***S*** (Figure 3b,e,h), the results of simple random sampling were lower than those of group analysis: $Mean\left(R_{C-1,k}\right)<Mean\left(R_{C-n,k}\right)<R_{T}$. Conversely, when ***R*** was negatively correlated with ***S*** (Figure 4c,f,i), the results of simple random sampling were higher than those of group analysis: $Mean\left(R_{C-1,k}\right)>Mean\left(R_{C-n,k}\right)>R_{T}$. As *n* increased, the results of $\mathrm{Mean}(R_{C-n,k})$ approached the actual $R_{T}$. The value of *k* had little effect on the average measured microbe/*Microcystis* value. However, for a given *n*, increasing *k* reduced $\mathrm{SD}(R_{C})$, indicating that an increased *k* could reduce the stochastic errors.

#### 3.3.2. The Distribution of *R_C_*

As Figure 4 shows, the number of individuals in each group (*n*) can affect the distribution of *R_C_*, which may be asymmetrically distributed. Therefore, we used permutation tests to compare two groups.

### 3.4. Permutation Test Between Two Populations

#### 3.4.1. Two Populations Had the Same Microbe/Microcystis Ratios

**Figure 5:**
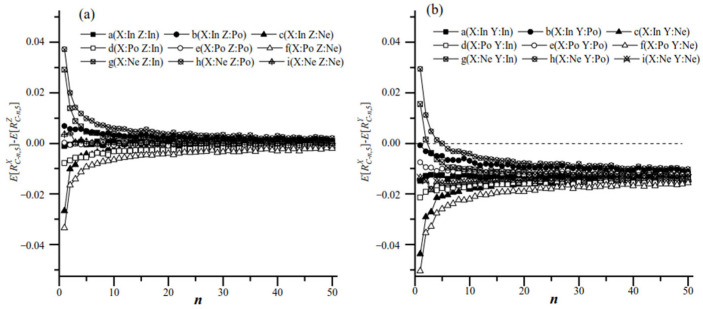
(**a**): The difference between $E\left[R_{C-n,5}^{X}\right]$ and $E\left[R_{C-n,5}^{Z}\right]$ calculated from two populations **X** and **Z** that had equal *R_T_* via sampling Plan(*n*,5). (**b**): The difference between $E\left[R_{C-n,5}^{X}\right]$ and $E\left[R_{C-n,5}^{Y}\right]$ calculated from two populations X and Y that had different *R_T_* via sampling Plan(*n*,5). “In” presents the ratio and size as independent. “Po” presents the ratio and size as positively correlated. “Ne” presents the ratio and size that were negatively correlated.

For populations with the same ratios (**X**, **Z**: $R_{T}^{X}=R_{T}^{Z}$), the correlation between the ratio and size could influence the value of *R_C_* (Figure 5a). When the ratio (***R***) and size (***S***) of **X** and **Z** were independent, $E\left[R_{C}^{X}\right]-E\left[R_{C}^{Z}\right]=E\left[R_{T}^{X}\right]-E\left[R_{T}^{Z}\right]=0$; however, when ***R*** and ***S*** in **X** or **Z** were correlated in either population, then $E\left[R_{C}^{X}\right]\neq E\left[R_{T}^{X}\right]$ or $E\left[R_{C}^{Z}\right]\neq E\left[R_{T}^{Z}\right]$. So $E\left[R_{C}^{X}\right]-E\left[R_{C}^{Z}\right]$ might not be zero. As the number of colonies in each group (*n*) increased, $E\left[R_{C}^{X}\right]-E\left[R_{C}^{Z}\right]$ decreased and approached zero.

**Figure 6:**
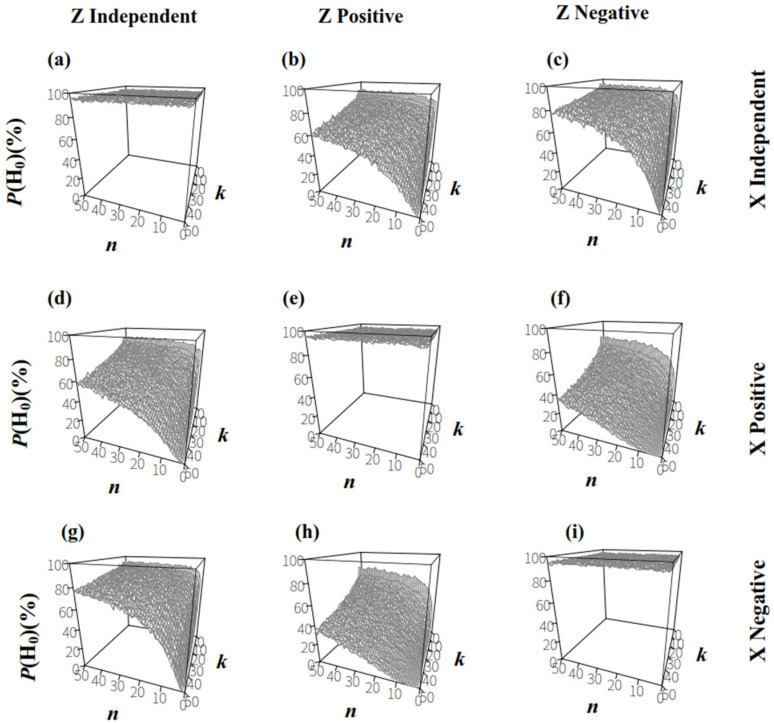
The proportion of accepting H_0_: $R_{T}^{X}=R_{T}^{Z}$ in permutation tests upon two populations that had same *R_T_*, (α = 0.05). (**a**): the sizes and ratios in population **X** and **Z** were both independent; (**b**): the sizes and ratios in population **X** were independent and in population **Z** were positively correlated; (**c**): the sizes and ratios in population **X** were independent and in population **Z** were negatively correlated; (**d**): the sizes and ratios in population **X** were positively correlated and in population **Z** were independent; (**e**): the sizes and ratios in population **X** and **Z** were both positively correlated; (**f**): the sizes and ratios in population **X** were positively correlated and in population **Z** were negatively correlated; (**g**): the sizes and ratios in population **X** were negatively correlated and in population **Z** were independent; (**h**): the sizes and ratios in population **X** were negatively correlated and in population **Z** were positively correlated; (**i**): the sizes and ratios in population **X** and **Z** were both negatively correlated.

For the comparison between populations **X** and **Z**, the null hypothesis (H_0_) was $R_{T}^{X}=R_{T}^{Z}$, and the alternative hypothesis (H_1_) was $R_{T}^{X}\neq R_{T}^{Z}$. When the sizes and ratios in populations **X** and **Z** were independent (Figure 6a), *P*(H_0_) was higher than 95% at a significance level of 0.05 for each sample plan, Plan(*n*,*k*). When the size and ratio in populations **X** or **Z** were correlated (Figure 6b–i), *P*(H_0_) decreased as *k* increased for a certain *n*; in contrast, for a given *k*, *P*(H_0_) increased as *n* increased. This suggested that increasing the number of individuals in sampling groups improved the testing accuracy of two populations with similar microbe/host ratios.

#### 3.4.2. Two Populations Had Different Microbe/Microcystis Ratios

For the populations with different microbe/*Microcystis* ratios at the population level ($R_{T}^{X}<R_{T}^{Y}$, such as *Xanthomonadales*: *M.a*. < *Xanthomonadales:M.w*. in this study), if the ratio and size in **X** or **Y** were correlated, then $\left|E\left[R_{C-n,5}^{X}\right]-E\left[R_{C-n,5}^{Y}\right]\right|\neq \left|R_{T}^{X}{-R}_{T}^{Y}\right|$. $E\left[R_{C}^{X}\right]-E\left[R_{C}^{Y}\right]$ could be either zero, positive or negative. As Figure 5b shows, when the number of colonies in each group (*n*) increased, $E\left[R_{C-n,5}^{X}\right]-E\left[R_{C-n,5}^{Y}\right]$ approached the true value of $R_{T}^{X}{-R}_{T}^{Y}$. When ***R*** and ***S*** were positively correlated or independent in **X**, as well as negatively correlated or independent in **Y**, $E\left[R_{C-n,5}^{X}\right]-E\left[R_{C-n,5}^{Y}\right]\leq R_{T}^{X}-E\left[R_{C-1,5}^{Y}\right]\leq R_{T}^{X}-R_{T}^{Y}<0$. Conversely, when ***R*** and ***S*** were negatively correlated in **X** or positively correlated in **Y**, $E\left[R_{C}^{X}\right]-E\left[R_{C}^{Y}\right]$ with an insufficient *n* might be positive, which is opposite to the actual situation.

**Figure 7:**
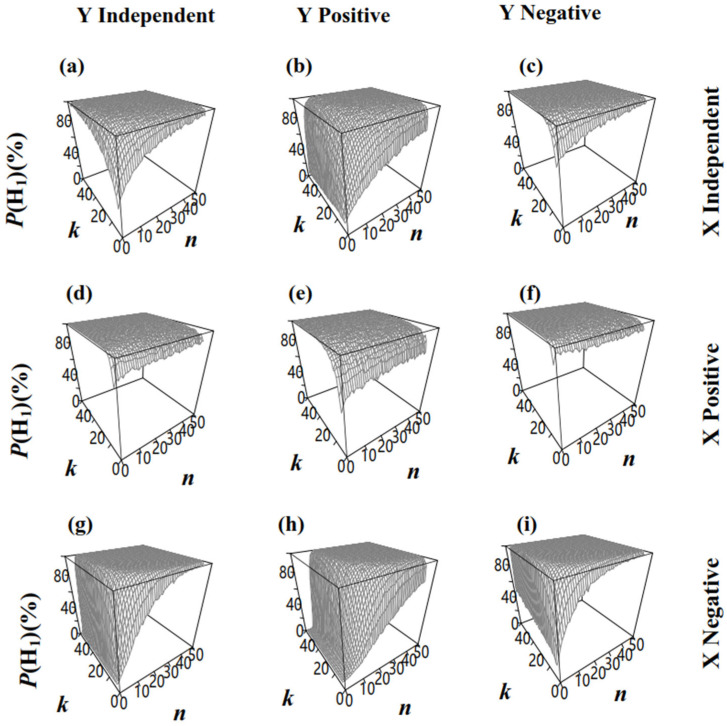
The proportion of accepting H_1_: $R_{T}^{X}<R_{T}^{Y}$ in permutation tests upon two populations that had different *R_T_* via sampling Plan(*n*,*k*) ($R_{T}^{X}<R_{T}^{Y}$, α = 0.05). (**a**): the sizes and ratios in population **X** and **Z** were both independent; (**b**): the sizes and ratios in population **X** were independent and in population **Z** were positively correlated; (**c**): the sizes and ratios in population **X** were independent and in population **Z** were negatively correlated; (**d**): the sizes and ratios in population **X** were positively correlated and in population **Z** were independent; (**e**): the sizes and ratios in population **X** and **Z** were both positively correlated; (**f**): the sizes and ratios in population **X** were positively correlated and in population **Z** were negatively correlated; (**g**): the sizes and ratios in population **X** were negatively correlated and in population **Z** were independent; (**h**): the sizes and ratios in population **X** were negatively correlated and in population **Z** were positively correlated; (**i**): the sizes and ratios in population **X** and **Z** were both negatively correlated.

For the significance testing of the two different groups ($R_{T}^{X}<R_{T}^{Y}$), the null hypothesis (H_0_) was $R_{T}^{X}=R_{T}^{Y}$, while the alternative hypothesis included H_1_ ($R_{T}^{X}<R_{T}^{Y}$) and H_2_ ($R_{T}^{X}>R_{T}^{Y}$). The effect of *n* on $P(H_{1})$ depended on the relationships between ***R*** and ***S***. $P(H_{1})$ increased with a larger *n* (Figure 7a–e,g–i), except when ***R*** and ***S*** were positively correlated in **X** and negatively correlated in **Y** (Figure 7f), where the probability of accepting the correct hypothesis (H_1_: $R_{T}^{X}<R_{T}^{Y}$) may decrease with a larger *n*. Considering that $P(H_{1})$ exceeded 0.95 under this situation (Figure 7f, $E\left[R_{C-n,5}^{X}\right]-E\left[R_{C-n,5}^{Y}\right]\leq R_{T}^{X}-E\left[R_{C-1,5}^{Y}\right]\leq R_{T}^{X}-R_{T}^{Y}<0$), it is unlikely to draw an incorrect conclusion due to the decreased $P(H_{1})$. In addition, when *n* is insufficient, increasing *k* may lead to a decrease in accuracy. Taking Figure 7h as an example, when *n* = 4, increasing *k* from 4 to 50 reduces the accuracy from 1.5% to 0.5%. In contrast, when *n* = 6, increasing *k* from 4 to 50 raises the accuracy from 3.9% to 19%. Therefore, if *n* is insufficient to correct the bias in $E\left[R_{C}^{X}\right]-E[R_{C}^{Y}]$ caused by SRS, increasing *n* can enhance the accuracy. On the other side, the group number *k* is also crucial for both precision and statistical power. As shown in Figure 7h, under the condition of *n* × *k* = 100, Plan(10,10), Plan(20,5), and Plan(25,4) all have sufficient *n* to correctly estimate $E\left[R_{C}^{X}\right]-E[R_{C}^{Y}]$, achieving accuracy of 32%, 44%, and 42%, respectively. This suggests that a small *k* may reduce the statistical power. In conclusion, for a given situation, such as our simulated dataset, there is an optimal sampling plan, Plan(n,k), under limited *n* × *k*, that maximizes statistical power.

## 4. Discussion

### 4.1. Effects of Sampling Plans on the Detection of Associated Microbes in Populations

If the ratio of microbes to *Microcystis* is correlated with the size of *Microcystis* colonies, then the average ratio calculated via simple random sampling may deviate from the exact population level ratio. Taking this into consideration, the present study suggests that group analysis is an appropriate method of reducing this deviation. In group analysis, *n* individuals are combined and treated as a testing group. Specifically, the collection of microbes through the filtration of a certain volume of water samples (which is widely used in plankton research) can also be considered as detecting several groups containing thousands of individuals. Testing *k* groups with randomly sampled individuals in each would obtain more accurate estimates than those calculated via simple random sampling.

Furthermore, the number of individuals in each group (*n*) can affect the distribution of *R_C_*, which may be asymmetrically distributed. Therefore, the t test and Wilcoxon test, which require symmetric distribution, are not suitable for comparing the associated microbes in two populations. Instead, permutation tests are used. For the permutation test of two populations, the number of distinct possible outcomes for the statistic is given by $\left(2k\right)!/(2!{\left(k!\right)}^{2})$ [42]. When the number of parallels is four, five, and six, the distinct possible outcomes are 35, 126, and 462, respectively. The precision of *p* values obtained from calculating the accurate distribution is 0.028, 0.008, and 0.002, respectively. Anderson [43] pointed out that when calculating the approximate distribution through random simulation, a significance level of 0.05 requires a 1000-time replacement, while a significance level of 0.01 requires a 5000-time replacement. Therefore, at least five parallels should be ensured to obtain a reliable result from comparing two populations.

The comparison of the ratios between two populations was not directly based on the exact ratio in populations (*R_T_*) but based on the calculation from sampled groups (*R_C_*). The number of individuals in each group (*n*) is crucial for enhancing the accuracy of *R_C_*, while the number of groups (*k*) could only decrease the stochastic error. If $E\left[R_{C}^{X}\right]-E[R_{C}^{Z}]$ differs from $R_{T}^{X}{-R}_{T}^{Z}$ and *n* is insufficient to correct the deviation between the sampling results and the actual values in the population, the likelihood of accepting the indirect results may increase with the number of test groups. This helps explain why, regardless of whether the ratio and size are correlated, increasing the number of individuals in each group can effectively enhance the accuracy of tests, whereas increasing the number of groups may reduce accuracy. Furthermore, practical surveys are usually constrained by *n* × *k*. For a particular community, there might be a best Plan(*n*,*k*) that provides the highest accuracy and statistical power under a limited *n* × *k*. However, most of the microbial communities in situ remained unknown. This resulted in being able to typically only choose to increase either *n* or *k*. Therefore, it is more advantageous to increase the number of individuals in each group rather than taking the risk of reducing accuracy by distributing the limited number of individuals across more testing groups.

### 4.2. Cost and Efficiency of Different Sampling Plans

There are two costs related to testing the microbes associated with *Microcystis* colonies: (1) sampling cost, which is approximately linearly related to the total number of individuals (N=n×k); and (2) the biological detection of each group, which is linearly related to *k*. The total cost of the isolation test is represented as follows:

(9) $$Cost=AN+Bk=AN+B\frac{N}{n}=\left(A+B\frac{1}{n}\right)N=Ank+Bk=\left(An+B\right)k$$

where A and B are constants.

For a given total number of individuals *N*, increasing *n* can reduce the biological testing costs, thereby lowering the overall experiment cost. Group analysis is less expensive than simple random sampling for testing the same number of individuals in the population.

### 4.3. Effects of Sampling Plans on the Limit of Detection

The limit of detection (LOD) is defined as the lowest quantity of a substance that can be distinguished from its absence. In a *Microcystis* population, if a testing group contains a colony with associated microbes exceeding the LOD, then this group can be detected. Moreover, other colonies within this group also contribute to the final result, regardless of whether their associated microbes are below or above the LOD. On the other side, if a group cannot be detected, it indicates that the associated microbes of each colony in this group are below the LOD.

Let *p* represent the proportion of colonies (marked as A) with associated microbes that are above the LOD; then, 1 − *p* represents the proportion of colonies (marked as B) with associated microbes that are below the LOD. The proportion of groups below the LOD is marked as *P_L_*; the proportion of groups where each colony falls below the LOD is marked as *P_B_.* Since some groups may be undetectable at the individual colony level but be detected as a group, then $P_{L}\leq P_{B}$. The proportion of colonies expected to contribute to the results via group analysis was *P_G_*, and via simple random sampling, it was *P_S_* (*P_S_ = p*). Then,

(10) $$P_{G}=1-P_{L}\geq 1-P_{B}=1-{\left(1-p\right)}^{n}\geq 1-\left(1-p\right)=p=P_{S}\;$$

Thus, the proportion of colonies contributing to the results is higher via group analysis than via simple random sampling, allowing group analysis to reflect more information. For instance, when only 5% of the colonies are detectable via simple random sampling (i.e., *p* = 0.05), then Plan(10,5) is expected to detect more than 40% of the colonies (*P_G_* ≥ 1 − (1 − 0.05)^10^), and Plan(50,5) is expected to detect over 92% (*P_G_* ≥ 1 − (1 − 0.05)^50^).

In addition, if small colonies or associated microbes are lower than the LOD, then they cannot be detected via simple random sampling, leading to biased results. In contrast, group analysis can provide more information about small colonies and those associated with a small number of microbes, resulting in higher accuracy compared to simple random sampling.

## 5. Conclusions

Some methods for detecting microplankton individuals primarily provide the ratio of objects to references, such as the ratio of 16S rDNA amplicons of associated microbes to *Microcystis* in this survey. If the ratio is correlated with the number of references (marked as the size of *Microcystis* colonies in this study), then the average ratio calculated via simple random sampling will deviate from the true population level ratio. Group analysis is an effective method for minimizing this deviation. Testing *k* groups that each contain *n* individuals is more accurate than calculations made through simple random sampling. Additionally, group analysis can reduce detection costs and lessen the impact of detection limits. It also enhances the accuracy of population comparisons. For comparing associated microbes between two populations, the detection of each population should include at least five groups, with each group containing as many individuals as possible.

## Figures and Tables

**Table 1:** The distribution and correlation of ***S*** and ***R*** in datasets.

Dataset	Sub-Dataset	R_T_	Pearson Correlation Between *R* and *S*		Lilliefors Test Ln(*S*)		Lilliefors Test Ln(*R*)	
			*R*	*P*	*D*	*P*	*D*	*P*
X	Sub-dataset 1	0.0108	−0.0008	0.5863	0.0009	0.4058	0.0011	0.1372
	Sub-dataset 2	0.0108	0.9463	0.0000 **			0.0010	0.2327
	Sub-dataset 3	0.0108	−0.2671	0.0000 **			0.0012	0.0640
Y	Sub-dataset 1	0.0238	−0.0007	0.6069	0.0013	0.0949	0.0010	0.2704
	Sub-dataset 2	0.0238	0.7655	0.0000 **			0.0011	0.1194
	Sub-dataset 3	0.0238	−0.3464	0.0000 **			0.0011	0.1288
Z	Sub-dataset 1	0.0108	−0.0008	0.5863	0.0009	0.4058	0.0011	0.1372
	Sub-dataset 2	0.0108	0.8239	0.0000 **			0.0009	0.3640
	Sub-dataset 3	0.0108	−0.2549	0.0000 **			0.0013	0.0839

“**”: *P* < 0.01.

## Data Availability

R codes and files needed to reproduce the analysis are available at https://github.com/LizLin29/GroupSample_test/tree/data (accessed on 17 February 2025) and https://github.com/LizLin29/GroupSample_test/tree/main (accessed on 13 February 2025).
